# A retrospective comparison between digital to conventional drainage systems for secondary spontaneous pneumothorax related to diffuse interstitial lung disease

**DOI:** 10.1111/crj.13654

**Published:** 2023-06-21

**Authors:** Kohei Shikano, Mitsuhiro Abe, Ryutaro Hirama, Shinsuke Kitahara, Kanae Maruyama, Dai Horiuchi, Noriko Sakuma, Daisuke Ishii, Takeshi Kawasaki, Hidenori Nakamura, Takuji Suzuki

**Affiliations:** ^1^ Department of Respirology, Graduate School of Medicine Chiba University Chiba Japan; ^2^ Department of Pulmonary Medicine Seirei Hamamatsu General Hospital Shizuoka Japan

**Keywords:** chest tubes, drainage, interstitial lung disease, interstitial pneumonia, pneumothorax

## Abstract

**Introduction:**

Secondary spontaneous pneumothorax (SSP) occurs as one of the complications associated with interstitial pneumonia (IP). Chest drainage is performed when there is a large volume of air in the pleural space. Notably, SSP with IP (SSP‐IP) is frequently not curable by chest drainage only. A digital drainage system (DDS) provides an objective evaluation of air leakage and maintains a pre‐determined negative pressure, compared to an analog drainage system (ADS). Few studies have reported the effectiveness of DDS in the treatment of SSP‐IP. This study aimed to assess the usefulness of DDS for SSP‐IP.

**Methods:**

This retrospective study included patients with SSP‐IP who had undergone chest drainage. We reviewed the included patients' medical records, laboratory data, computed tomography findings, and pulmonary function data.

**Results:**

DDS was used in 24 patients and ADS in 49 patients. The mean duration of chest drainage was 11.4 ± 1.9 days in the DDS group and 14.2 ± 1.3 days in the ADS group, which was not significantly different (*p* = 0.218). Surgery, pleurodesis, and/or factor XIII administration were performed in 40 patients. Additionally, five (20.8%) patients in the DDS group and nine (18.4%) in the ADS group had a recurrence of pneumothorax within 4 weeks (*p* = 1.000). One patient (14%) in the DDS group and six (12.2%) in the ADS group (*p* = 0.414) were cured of pneumothorax but later died.

**Conclusion:**

DDS did not demonstrate a significant difference in the shortening of chest drainage duration. Further study is needed to validate the results of this study.

## Graphical abstract

Digital drainage systems are used for treating pneumothorax associated with interstitial pneumonia. We conducted a retrospective study of patients who received chest drainage and found that digital drainage systems shorten the chest drainage duration. Digital drainage systems might be useful for the treatment of pneumothorax associated with interstitial pneumonia.

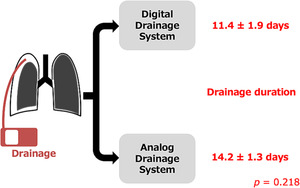

## 1 INTRODUCTION

Pneumothorax is defined as the presence of air in the pleural space between the lung and chest wall.^1^ Secondary spontaneous pneumothorax (SSP) occurs as a complication of various lung diseases and is often experienced in daily clinical practice. Interstitial pneumonia (IP) is one of the lung diseases causing SSP, with risk factors including the presence of computed tomography (CT) abnormalities, lower vital capacity, lower body mass index, receiving long‐term oxygen therapy, and methylprednisolone pulse therapy.^2^, ^3^, ^4^ Additionally, SSP with IP (SSP‐IP) has been reported to occur in 20.2% of patients with idiopathic pulmonary fibrosis (IPF) and is considered a poor prognostic factor.^5^

Chest drainage should be performed for patients with pneumothorax affecting a large area of the pleural space.^6^, ^7^ Notably, SSP‐IP is frequently not curable by chest drainage only. Surgery is one of the most common additional treatments and is the first choice for pneumothorax not cured by chest drainage alone.^6^ However, SSP‐IP often recurs even after surgery, and the postoperative mortality rate is high as 2.9–4.1%.^8^, ^9^, ^10^ Furthermore, some patients with SSP‐IP do not tolerate surgery under general anesthesia. Therefore, patients with SSP‐IP tend to avoid surgery and receive alternative treatments. A previous study reported that 38.1% of cases of SSP‐IP required alternative treatments such as pleurodesis in addition to chest drainage.^11^

A digital drainage system (DDS), Thopaz® (Medela), has been introduced into clinical practice for use in chest drainage. Compared to a conventional analog drainage system (ADS), this system enables objective evaluation of air leaks and maintains a pre‐determined negative pressure without the influence of position changes or obstruction of tubes.^12^, ^13^, ^14^ In addition, the ability to continuously record air leaks reduces variability by physicians in the timing of drain removal.^15^, ^16^ This DDS was expected to shorten the drainage and hospitalization periods and hospitalization costs. Several studies reported the effectiveness of DDS in spontaneous pneumothorax or after thoracic surgery.^13^, ^17^, ^18^ On the other hand, only one case report has reported its effectiveness in the treatment of SSP‐IP.^19^ Therefore, this study aimed to assess the usefulness of DDS for SSP‐IP.

## 2 MATERIALS AND METHODS

### 2.1 Study design and patients

We conducted a retrospective chart review of patients with SSP‐IP who had undergone chest drainage at the Chiba University Hospital or the Seirei Hamamatsu General Hospital between April 2016 and March 2022. The exclusion criteria were as follows: (1) patients aged <20 years, (2) patients who died with uncured pneumothorax, and (3) patients whose drainage system was changed within 48 h after chest drainage.

All analyses were performed in accordance with the amended Declaration of Helsinki. Written informed consent for chest drainage was obtained from each patient. Data anonymization and privacy issues were strictly addressed. The study protocol was approved by the Human Ethics Committee of our institution (approval number M10306), and individual consent for this retrospective analysis was waived.

### 2.2 Clinical data collection

We reviewed the included patients' medical records, laboratory data, CT findings, and pulmonary function data. The CT images were reviewed by two pulmonologists, and the findings were classified into fibrosis, cysts, fibrosis and cysts, and others. Pulmonary function data were obtained within 1 year of pneumothorax onset.

### 2.3 Treatment of pneumothorax

The management of chest drain depends on a decision made by each clinician. After chest drainage, the chest tube was connected to the DDS or ADS. The intrathoracic pressure was between −8 cm H_2_O and −40 cm H_2_O in the DDS group and 0 cm H_2_O and −20 cm H_2_O in the ADS group.

The chest tube was removed when an air leak was not detected and lung expansion on the chest X‐ray was obtained sufficiently. No air leak was set at lower than 30 mL/min of airflow in DDS and as a lack of an air bubble in ADS. In many cases, the clump test was performed to check that the chest tube was clumped, and the lung was not collapsed.

When pneumothorax was not cured by chest drainage alone, other additional treatments, including surgery, were performed based on the patient's condition and the clinical course.

### 2.4 Statistical analysis

The median and range of continuous data were calculated. Counts and percentages were determined for categorical data. Continuous data were analyzed using the Wilcoxon rank‐sum test and categorical data were analyzed using the Fisher's exact test. Two‐sided *p* values of <0.05 were considered statistically significant. All analyses were performed using SAS software v.9.4 for Windows (SAS Institute Inc., Cary, NC, USA) and JMP pro 16.0.0 software (SAS Institute Inc. Cary, NC, USA).

## 3 RESULTS

### 3.1 Study population and characteristics

**FIGURE 1:**
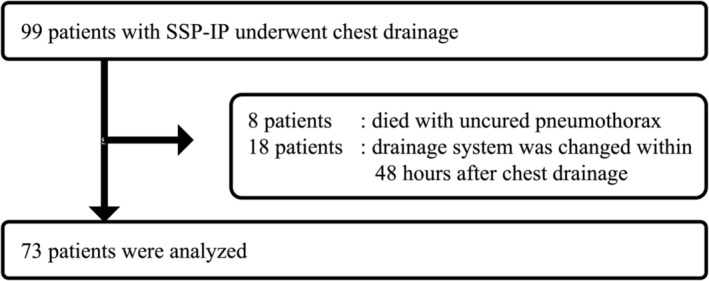
Flow diagram for study participants.

In total, 99 patients with SSP‐IP underwent chest drainage. Of these, 26 patients were excluded according to the criteria, and a total of 73 patients were included in the study (Figure 1). Patient characteristics are summarized in Table 1. Digital drainage was performed in 24 (32.9%) patients and analog drainage in 49 (67.1%) patients. There were 49 (67.1%) males and 24 (32.9%) females, with a mean age of 65.3 years. The CT image showed fibrosis in 52 (71.2%) cases and cysts in 42 (57.5%) cases. Prednisolone was administrated for 21 (28.8%) patients and anti‐fibrotic medication for 16 (21.9%) patients for the treatment of IP. Regarding pneumothorax conditions, 52 (71.2%) cases were first episode of pneumothorax, and the right and left sides were affected in almost half cases each. Significant differences were observed in age and CT findings between the DDS and ADS groups.

**TABLE 1:** Patient characteristics.

	Total	Digital drainage	Analog drainage	*p* value
Total	73	24	49	
Sex				
Male/Female, *n*	48/25	15/9	33/16	0.794
Age, years	65.3 ± 1.8	71.7 ± 3.0	62.1 ± 2.1	**0.012**
CT findings				
Fibrosis/cyst/fibrosis and cyst, *n*	26/16/26	11/1/11	15/15/15	**0.036**
IPF	18	6	12	1.000
Treatments of IP				
Prednisolone, *n*	ADDIN [char_align:decimal‐dot] \* MERGEFORMAT 21	6	15	0.785
Antifibrotic drugs, *n*	16	4	12	0.555
Long‐term oxygen therapy, *n*	27	11	16	0.310
Pulmonary function tests				
%VC, %predicted	59.9 ± 4.0	55.5 ± 7.6	61.7 ± 4.8	0.491
FEV1, %predicted	62.6 ± 4.0	62.8 ± 7.7	62.6 ± 4.9	0.982
Pneumothorax conditions				
First/Recurrent, *n*	52/21	18/6	34/15	0.785
Right/Left, *n*	38/35	11/13	27/22	0.619

Abbreviations: IP, interstitial pneumonia; IPF, idiopathic pulmonary fibrosis; FEV1 forced expiratory volume in 1 s; VC vital capacity.

### 3.2 Treatments and outcomes of pneumothorax

Table 2 shows the treatments and outcomes of pneumothorax. The mean duration of drainage tended to be shorter in the DDS group than in the ADS group; however, the difference was not significant (11.4 ± 1.9 days in the DDS group vs. 14.2 ± 1.3 days in the ADS group, *p* = 0.218). Surgery, pleurodesis, and/or factor XIII administration were performed for treating pneumothorax in 40 (54.8%) patients. Five (20.8%) patients in the DDS group and nine (18.4%) in the ADS group had a recurrence of pneumothorax within 4 weeks (*p* = 1.000). One (4.2%) patient in the DDS group and six (12.2%) in the ADS group (*p* = 0.414) were cured of pneumothorax but died.

**TABLE 2:** Treatments and outcomes of pneumothorax in the digital and the analog drainage group.

	Total	Digital drainage	Analog drainage	*p* value
Total	73	24	49	
Duration of chest drainage, days	13.3 ± 1.1	11.4 ± 1.9	14.2 ± 1.3	0.218
Treatments of pneumothorax				
Any, *n*	40	14	26	0.803
Surgery, *n*	11	3	8	1.000
Pleurodesis, *n*	26	10	16	0.603
Factor XIII administration, *n*	5	2	3	1.000
Recurrence within 4 weeks, *n*	14	5	9	1.000
Death, *n*	7	1	6	0.414

### 3.3 Treatments and outcomes of pneumothorax in subgroups

The subgroup analysis was performed on the group of cases excluding surgery and those with IPF. Table 3 shows the results of the analysis of 62 patients, excluding those who underwent surgery. The mean duration of chest drainage was 11.4 ± 1.8 days in the DDS group and 13.4 ± 1.3 days in the ADS group, which was not significantly different (*p* = 0.369). There was no significant difference between the two groups in treatments, recurrence, and death. Eighteen cases with SSP with IPF were analyzed, and the results are summarized in Table 4. The mean duration of chest drainage was 11.3 ± 4.7 days in the DDS group and 13.8 ± 3.3 days in the ADS group. This result was also not significantly different (*p* = 0.668). Treatments, recurrence, and death showed no significant differences between the two groups.

**TABLE 3:** Treatments and outcomes of pneumothorax excluding surgery cases in the digital and the analog drainage group.

	Total	Digital drainage	Analog drainage	*p* value
Total	62	21	41	
Duration of chest drainage, days	12.7 ± 1.1	11.4 ± 1.8	13.4 ± 1.3	0.369
Treatments of pneumothorax				
Any, *n*	29	11	18	0.597
Pleurodesis, *n*	26	10	16	0.592
Factor XIII administration, *n*	5	2	3	1.000
Recurrence within 4 weeks, n	11	5	6	0.485
Death, *n*	7	1	6	0.406

**TABLE 4:** Treatments and outcomes of pneumothorax with idiopathic pulmonary fibrosis in the digital and the analog drainage group.

	Total	Digital drainage	Analog drainage	*p* value
Total	18	6	12	
Duration of chest drainage, days	13.0 ± 2.6	11.3 ± 4.7	13.8 ± 3.3	0.668
Treatments of pneumothorax				
Any, *n*	12	5	7	0.600
Surgery, *n*	3	1	2	1.000
Pleurodesis, *n*	9	4	5	0.620
Factor XIII administration, *n*	1	0	1	1.000
Recurrence within 4 weeks, *n*	3	1	2	0.485
Death, *n*	1	0	1	1.000

## 4 DISCUSSION

This is the first study to report a comparison of DDS and ADS for treating SSP‐IP in multiple patients, and the duration of chest drainage was approximately 3 days shorter with DDS, although the difference was not statistically significant. Lung compliance in patients with interstitial pneumonia decreases due to the destruction of alveolar architecture,^20^, ^21^ and thus, full expansion cannot be achieved in some cases. Additionally, continued suctioning may lead to lung injury. DDS provides a quantitative recording of air leakage,^13^ making it easier to determine whether pneumothorax is cured, even if the full expansion is not obtained. Additionally, DDS can automatically maintain suction pressure without the influence of positioning or obstruction of the chest drain.^13^ This helps avoid excessive suctioning and thus, lung injury may be less likely to occur. These may explain the difference in the chest drainage duration.

However, this study did not show any significant differences between DDS and ADS for the treatment of SSP‐IP. One reason for this result could be due to the different characteristics of the patients. In this study, the details of CT findings differed between the DDS and ADS groups. The ADS group may have had a more complicated disease than the digital drainage system group, because of more use of prednisolone and/or antifibrotic drugs. Both fibrosis and cysts, the latter of which is represented by chronic obstructive pulmonary disease, cause lung fragility.^22^ However, lung compliance differs between fibrosis and cysts; therefore, the different characteristics of the patients might lead to no significant differences between DDS and ADS for the treatment of SSP‐IP. Another reason is the lack of criteria for clamping the chest tube before chest drain removal. Although clamping is unnecessary in cases of DDS for providing quantitative air leak,^16^ clamping was performed in some cases with DDS in this study. Therefore, a study in which these factors are aligned should be performed.

In cases with SSP and IPF, the chest drainage duration tended to be shorter in the DDS group. Pneumothorax is a poor prognostic factor of IPF. Nishimoto et al reported that 20.2% of patients with IPF developed pneumothorax, and the median survival time from the onset of SSP was 13.3 months.^5^ Although it is not clear whether shortening the drainage duration contributes to improved survival, it may prevent deterioration of the patients' general condition. Since the number of cases included in this study is small, analysis of a larger population is needed.

In the current study, additional treatments, surgery, and/or pleurodesis, were performed as much as previously reported.^11^ Although surgery should be sought in cases of persistent air leakage,^6^ it tends to be avoided for pneumothorax with IP because of the possibility of causing acute exacerbation of IP.^23^, ^24^ However, previous reports indicated that surgery could be performed safely in such cases.^25^ Pleurodesis, which is recommended as an alternative treatment in cases where surgery cannot be performed,^1^, ^6^ also has the risk of acute exacerbation of IP and has a higher rate of the recurrence of pneumothorax than surgery.^25^ Therefore, these treatment choices should be made with careful consideration. Porcel recommends that studies comparing treatments for SSP‐IP are needed.^26^ In the present study, patients received various treatments for SSP‐IP, and the drainage duration was shorter in the DDS group than in the ADS group, with or without surgery. In addition, there were no deaths among patients who underwent surgery. Indicating what treatment should be performed is beyond the scope of the present study. However, in any case, it is important to perform appropriate multidisciplinary treatment, including surgery, in combination with DDS.

Prolonged chest drainage may cause complications such as pain, intrapleural infection, and drain‐related visceral injury,^27^ contributing to the deterioration of the patient's condition. These events lead to a decline in activities of daily living, prolonged hospitalization, and an increase in medical costs.^28^

There are some limitations to our study. First, this was a retrospective study. Second, the severity of IP varied in this study. The CT findings of fibrosis are different between the ADS and DDS groups, which is a major limitation of this study. Surgery, which is an invasive procedure may have been avoided for the severe cases of IP. A prospective, randomized, multicenter study with a large sample size should be performed. Finally, this study did not analyze the length of hospital stay and cost‐effectiveness. These items should be analyzed together in future studies. Needless to say, the patients' backgrounds must be aligned so as not to affect the length of hospital stay.

In conclusion, the current study aimed to assess the usefulness of DDS for SSP‐IP by retrospectively analyzing data of patients who received chest drainage. The DDS group did not demonstrate a significant difference in the shortening of chest drainage duration. Further studies are needed that multicenter prospective studies with aligned patient backgrounds.

## AUTHOR CONTRIBUTIONS

Kohei Shikano, Mitsuhiro Abe, and Takuji Suzuki contributed to the study concept and design. Kohei Shikano, Ryutaro Hirama, and Shinsuke Kitahara examined the enrolled patients in the hospital. In addition, Kohei Shikano performed the statistical analyses. All authors have read and approved the final manuscript.

## CONFLICT OF INTEREST STATEMENT

All authors have completed the ICMJE uniform disclosure form. The authors have no conflicts of interest to declare.

## ETHICAL APPROVAL STATEMENT/PATIENT CONSENT STATEMENT

The authors are accountable for all aspects of the work in ensuring that questions related to the accuracy or integrity of any part of the work are appropriately investigated and resolved. The study was performed in accordance with the amended Declaration of Helsinki. Written informed consent for chest drainage was obtained from each patient. Data anonymization and privacy issues were strictly addressed. The study protocol was approved by the Human Ethics Committee of Chiba University Hospital (approval number M10306) and individual consent for this retrospective analysis was waived.

## Data Availability

Derived data supporting the findings of this study are available from the corresponding author on request.

## References

[1] Bintcliffe O , Maskell N . Spontaneous pneumothorax. BMJ. 2014;348(may08 1):g2928. doi:10.1136/bmj.g2928 24812003

[2] Tsukahara Y , Yamakawa H , Tsumiyama E , et al. Clinical features and prognosis of secondary pneumothorax in pulmonary emphysema, interstitial pneumonia, and combined pulmonary fibrosis and emphysema. Ann Japan Respir Soc. 2020;9:160‐165.

[3] Iwasawa T , Ogura T , Takahashi H , et al. Pneumothorax and idiopathic pulmonary fibrosis. Jpn J Radiol. 2010;28(9):672‐679. doi:10.1007/s11604-010-0494-1 21113751

[4] Nishimoto K , Fujisawa T , Yoshimura K , et al. Pneumothorax in connective tissue disease‐associated interstitial lung disease. PLoS ONE. 2020;15(7):e0235624. doi:10.1371/journal.pone.0235624 32634173 PMC7340294

[5] Nishimoto K , Fujisawa T , Yoshimura K , et al. The prognostic significance of pneumothorax in patients with idiopathic pulmonary fibrosis. Respirology. 2018;23(5):519‐525. doi:10.1111/resp.13219 29130562

[6] MacDuff A , Arnold A , Harvey J , BTS Pleural Disease Guideline Group . Management of spontaneous pneumothorax: British Thoracic Society pleural disease guideline 2010. Thorax. 2010;65 Suppl 2(Suppl 2):ii18‐ii31. doi:10.1136/thx.2010.136986 20696690

[7] Baumann MH , Strange C , Heffner JE , et al. AACP pneumothorax consensus group. Management of spontaneous pneumothorax: an American College of Chest Physicians Delphi consensus statement. Chest. 2001;119(2):590‐602. doi:10.1378/chest.119.2.590 11171742

[8] Kawai N , Kawaguchi T , Yasukawa M , Tojo T , Sawabata N , Taniguchi S . Surgical treatment for secondary spontaneous pneumothorax: a risk factor analysis. Surg Today. 2021;51(6):994‐1000. doi:10.1007/s00595-020-02206-0 33483786

[9] Igai H , Kamiyoshihara M , Ibe T , Kawatani N , Shimizu K . Surgical treatment for elderly patients with secondary spontaneous pneumothorax. Gen Thorac Cardiovasc Surg. 2016;64(5):267‐272. doi:10.1007/s11748-016-0636-1 26961341

[10] Isaka M , Asai K , Urabe N . Surgery for secondary spontaneous pneumothorax: risk factors for recurrence and morbidity. Interact Cardiovasc Thorac Surg. 2013;17(2):247‐252. doi:10.1093/icvts/ivt221 23674562 PMC3715204

[11] Yamazaki R , Nishiyama O , Gose K , et al. Pneumothorax in patients with idiopathic pulmonary fibrosis: a real‐world experience. BMC Pulm Med. 2021;21(1):5. doi:10.1186/s12890-020-01370-w 33407311 PMC7789641

[12] Dernevik L , Belboul A , Rådberg G . Initial experience with the world's first digital drainage system. The benefits of recording air leaks with graphic representation. Eur J Cardiothorac Surg. 2007;31(2):209‐213. doi:10.1016/j.ejcts.2006.10.038 17194600

[13] Zhou J , Lyu M , Chen N , et al. Digital chest drainage is better than traditional chest drainage following pulmonary surgery: a meta‐analysis. Eur J Cardiothorac Surg. 2018;54(4):635‐643. doi:10.1093/ejcts/ezy141 29659768

[14] Arai H , Tajiri M , Kameda Y , et al. Evaluation of a digital drainage system (Thopaz) in over 250 cases at a single site: a retrospective case‐control study. Clin Respir J. 2018;12(4):1454‐1459. doi:10.1111/crj.12683 28776940

[15] Varela G , Jiménez MF , Novoa NM , Aranda JL . Postoperative chest tube management: measuring air leak using an electronic device decreases variability in the clinical practice. Eur J Cardiothorac Surg. 2009;35(1):28‐31. doi:10.1016/j.ejcts.2008.09.005 18848460

[16] Anegg U , Lindenmann J , Matzi V , et al. AIRFIX: the first digital postoperative chest tube airflowmetry—a novel method to quantify air leakage after lung resection. Eur J Cardiothorac Surg. 2006;29(6):867‐872. doi:10.1016/j.ejcts.2006.03.026 16675248

[17] Lee SA , Kim JS , Chee HK , et al. Clinical application of a digital thoracic drainage system for objectifying and quantifying air leak versus the traditional vacuum system: a retrospective observational study. J Thorac Dis. 2021;13(2):1020‐1035. doi:10.21037/jtd-20-2993 33717575 PMC7947544

[18] Yagi S , Miwa H , Kono M , et al. Comparison of clinical utility between digital and analog drainage systems in patients with spontaneous pneumothorax. Respir Investig. 2022;60(6):840‐846. doi:10.1016/j.resinv.2022.06.013 35965216

[19] Jenkins WS , Hall DP , Dhaliwal K , Hill AT , Hirani N . The use of a portable digital thoracic suction Thopaz drainage system for the management of a persistent spontaneous secondary pneumothorax in a patient with underlying interstitial lung disease. BMJ Case Rep. 2012;2012(jun07 1):bcr0220125881. doi:10.1136/bcr.02.2012.5881 PMC4543047 22684832

[20] Edwards Z , Annamaraju P . Physiology, lung compliance. StatPearls; 2022. 32119404

[21] Richeldi L , Collard HR , Jones MG . Idiopathic pulmonary fibrosis. Lancet. 2017;389(10082):1941‐1952. doi:10.1016/S0140-6736(17)30866-8 28365056

[22] Papandrinopoulou D , Tzouda V , Tsoukalas G . Lung compliance and chronic obstructive pulmonary disease. Pulm Med. 2012;2012:542769. doi:10.1155/2012/542769 23150821 PMC3486437

[23] Choi SM , Lee J , Park YS , et al. Postoperative pulmonary complications after surgery in patients with interstitial lung disease. Respiration. 2014;87(4):287‐293. doi:10.1159/000357046 24577160

[24] Amundson WH , Racila E , Allen T , et al. Acute exacerbation of interstitial lung disease after procedures. Respir Med. 2019;150:30‐37. doi:10.1016/j.rmed.2019.02.012 30961948

[25] Watanabe T , Tanahashi M , Suzuki E , et al. Treatment of secondary pneumothorax with interstitial lung disease: the surgical indications at the start of treatment is important. J Thorac Dis. 2022;14(5):1393‐1400. doi:10.21037/jtd-21-1851 35693624 PMC9186251

[26] Porcel JM . Secondary spontaneous pneumothorax in idiopathic pulmonary fibrosis: grim news. Respirology. 2018;23(5):448‐449. doi:10.1111/resp.13253 29316046

[27] Havelock T , Teoh R , Laws D , Gleeson F , BTS Pleural Disease Guideline Group . Pleural procedures and thoracic ultrasound: British Thoracic Society pleural disease guideline 2010. Thorax. 2010;65 Suppl 2(Suppl 2):i61‐i76. doi:10.1136/thx.2010.137026 20696688

[28] Plojoux J , Froudarakis M , Janssens JP , Soccal PM , Tschopp JM . New insights and improved strategies for the management of primary spontaneous pneumothorax. Clin Respir J. 2019;13(4):195‐201. doi:10.1111/crj.12990 30615303

